# Ectrodactyly, Cleft Lip/Palate, and Urinary Anomalies With a Tumor Protein p63 (TP63) Mutation: A Case Report and Literature Review

**DOI:** 10.7759/cureus.92888

**Published:** 2025-09-21

**Authors:** Rayan H Mohamed, Haydy M Khalifa, Hisham Y Hassan, Eman Shajira, Fahad A Al-Qashar

**Affiliations:** 1 Department of Pediatrics, Bahrain Defence Force Hospital, Royal Medical Services, Riffa, BHR; 2 Department of Cytogenetics, Banoon Assisted Reproduction Technology (ART) and Cytogenetics Centre, Bahrain Defence Force Hospital, Royal Medical Services, Riffa, BHR

**Keywords:** cleft lip and palate, ectrodactyly, ectrodactyly-ectodermal dysplasia-clefting syndrome, eec syndrome, gene mutation, tp63 gene

## Abstract

Heterozygous mutations in the tumor protein p63 (TP63) gene underlie a spectrum of autosomal dominant syndromes, affecting ectodermal, limb, and orofacial development. We report an infant born with ectrodactyly (split-hand/foot malformation), cleft lip and palate, and a solitary kidney with hydronephrosis. Genetic testing revealed a heterozygous TP63 missense variant, c.740A>G (p.His247Arg), inherited from his affected father, confirming Ectrodactyly-Ectodermal Dysplasia-Clefting (EEC) syndrome. This case highlighted the clinical significance of the TP63 p.His247Arg mutation, previously reported as pathogenic in EEC. The infant’s abnormalities required multidisciplinary management, including surgical, urologic, and nutritional support. Our findings emphasized the markedly variable expression associated with TP63-related disorders. The father, despite carrying the same mutation, exhibited only a milder clinical presentation. Early genetic diagnosis was crucial for tailored management and family counseling. This report underscores the importance of recognizing TP63 syndromes. It also reviews some relevant cases and studies from the literature and illustrates how genetic findings inform prognosis and guide comprehensive care in EEC syndrome.

## Introduction

The tumor protein p63 (TP63) gene, located on the long arm of chromosome 3 (3q28), encodes the p63 transcription factor, a critical regulator of embryonic development and epithelial stem cell maintenance [1,2]. P63 is a p53 family protein expressed in the basal layers of epithelia, with dual major isoforms (TAp63 and ΔNp63; TA isoforms=transactivation, ΔN isoforms=dominant negative roles). that have distinct roles in growth and differentiation [3]. Mouse models lacking p63 die at birth with profound limb truncations, craniofacial defects, and absent stratified epithelia, reflecting the gene’s essential function in limb morphogenesis and epidermal development [2,4]. In humans, heterozygous TP63 mutations cause a group of overlapping autosomal dominant syndromes collectively known as TP63-related disorders, characterized by variable combinations of ectodermal dysplasia, orofacial clefting, and limb malformations [5]. These include Ectrodactyly-Ectodermal Dysplasia-Cleft Lip/Palate (EEC) syndrome (clinically characterized by limb malformations such as ectrodactyly, ectodermal defects including sparse hair and dry skin, and orofacial clefting, which provide the clinical background for understanding the underlying genetic mechanisms), Ankyloblepharon-Ectodermal Defects-Cleft Lip/Palate (AEC or Hay-Wells syndrome), Rapp-Hodgkin syndrome, Acro-Dermo-Ungual-Lacrimal-Tooth (ADULT) syndrome, Limb-Mammary syndrome, and non-syndromic Split-Hand/Foot Malformation type 4 [1,6,7]. Notably, these diagnoses share overlapping features, which has led to their consideration as part of a phenotypic spectrum of one genetic pathway.

Patients with TP63 mutations typically present with some combination of ectodermal dysplasia, facial clefts, and limb malformations ranging from syndactyly to ectrodactyly [1,3]. Additional findings can include abnormalities of the lacrimal ducts, hypopigmented or fragile skin, breast and nipple hypoplasia, hearing loss, and urogenital anomalies such as hypospadias or renal malformations [8]. Because of the significant clinical overlap, a precise diagnosis often relies on molecular genetic testing [8]. Most EEC-causing mutations are missense changes in the DNA-binding domain of p63, leading to dominant-negative effects on wild-type p63 function [5]. In contrast, certain mutations, e.g., in the ADULT syndrome, act via gain-of-function mechanisms, illustrating genotype-phenotype correlations within TP63 disorders [9,10]. Despite the multi-system physical abnormalities, intellectual development is typically normal in these syndromes, which helps focus management on the physiological and reconstructive needs [11].

Early identification of a TP63-related syndrome is critical because it prompts comprehensive evaluation of associated anomalies and appropriate specialist referrals [7]. Approximately half of the EEC cases result from de novo mutations, whereas others are familial, as in this report [10,12]. We describe this case to underscore the importance of recognizing variable expressivity in TP63-related disorders, which has direct implications for diagnosis and genetic counseling.

This case report aims to describe a rare presentation of EEC syndrome associated with urinary tract anomalies, highlighting the clinical, radiological, and genetic findings, and to discuss its implications for diagnosis and patient management.

## Case presentation

**Figure 1:**
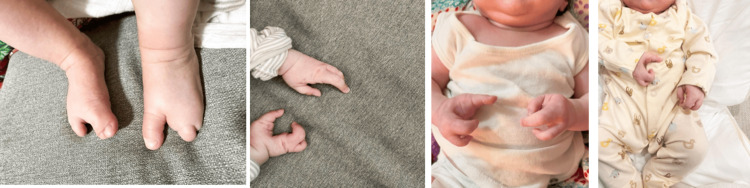
Key clinical findings included ectrodactyly involving both hands and feet, hypoplastic nails, and limb malformations

A six-month-old male infant was born with multiple congenital anomalies. He was the first child of non-consanguineous parents, although the father was noted to have features suggestive of an EEC spectrum disorder. The infant’s birth was at term via an uncomplicated delivery, but physical examination at birth revealed obvious malformations. He had a unilateral cleft lip on the left and a complete cleft palate involving both hard and soft palates. Both hands and feet exhibited central ray defects consistent with ectrodactyly (Figure 1).

Additionally, an abdominal ultrasound conducted shortly after birth due to palpable asymmetry revealed a right-sided solitary kidney.

Craniofacial examination showed a left-sided cleft lip repaired in the neonatal period and a persistent bilateral cleft of the palate. The scalp hair appeared sparse and fine, and there was mild xerosis of the skin, consistent with features of ectodermal dysplasia. The hands demonstrated a split-hand malformation: the median rays of each hand were absent, giving the appearance of a central cleft in the palms. The feet had a similar split configuration, accompanied by syndactyly of some remaining toes. A small sacral dimple was noted in the midline of the lower back. Neurologic exam was normal, with normal tone, strength, and intact primitive reflexes appropriate for age. Respiratory and cardiac examinations were unremarkable. The abdomen was soft with no organomegaly. The infant’s external genitalia were male and normal in appearance, with a normally formed penis and descended testes.

**Figure 2:**
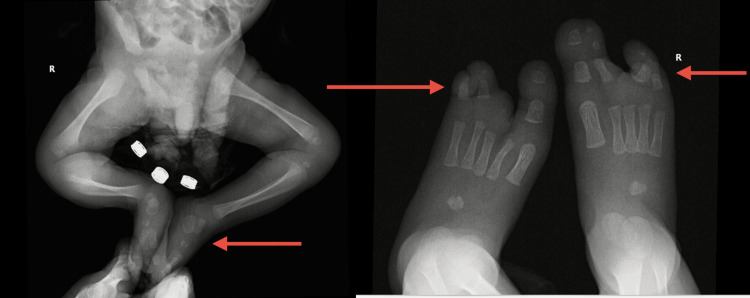
Anteroposterior radiograph of the lower limbs Images demonstrate bilateral talipes equinovarus with preserved hip joint alignment. Foot films reveal central ray deficiency consistent with ectrodactyly.

A comprehensive evaluation was undertaken to characterize the full extent of the infant’s anomalies. Skeletal survey X-rays of the limbs confirmed the bilateral split-hand and split-foot deformities with the absence of central digital rays, and fusion of some digits in the feet (Figure 2).

No other bony abnormalities were noted on the survey. Given the sacral dimple, a spine and sacral ultrasound was performed, which was normal and showed no evidence of spinal dysraphism. Cranial ultrasound imaging was also normal, with no intracranial abnormalities detected. Ophthalmologic examination and newborn hearing screening were normal, and repeat hearing assessment at three months of age remained within normal limits, indicating no early-onset hearing loss.

Recognizing the feeding challenges posed by the cleft palate, the care team involved a nutritionist and speech therapist early on. The multidisciplinary team planned for surgical repair of the cleft palate in infancy, and for hand reconstructive evaluations by plastic and orthopedic surgery with the goal of optimizing grasp function.

Given the constellation of ectrodactyly, ectodermal features, and orofacial clefting, a TP63-related disorder (EEC syndrome) was suspected. The infant underwent genetic testing, including chromosomal karyotyping (which showed a normal male, 46, XY), as well as whole exome sequencing. The whole exome sequencing identified a heterozygous missense variant in the TP63 gene: NM_003722.4: c.740A>G, resulting in a p.His247Arg amino acid substitution. This variant has been reported as pathogenic in the context of EEC syndrome. Targeted parental testing revealed that the father of the index case harbored the same mutation, despite no known family history of the condition, suggesting a possible de novo event in him. The mother was found to have a normal sequence at this locus. The father, now 28 years old, had a history of congenital hand and foot anomalies consistent with ectrodactyly and a repaired cleft lip in childhood, confirming that he was also affected by EEC syndrome. Thus, the genetic findings established an autosomal dominant inheritance in this family, with the pathogenic TP63 variant passed from father to son. Genetic counseling was provided to the family regarding their future pregnancies, and options such as preimplantation genetic testing via in vitro fertilization or prenatal diagnostic testing were discussed.

**Figure 3:**
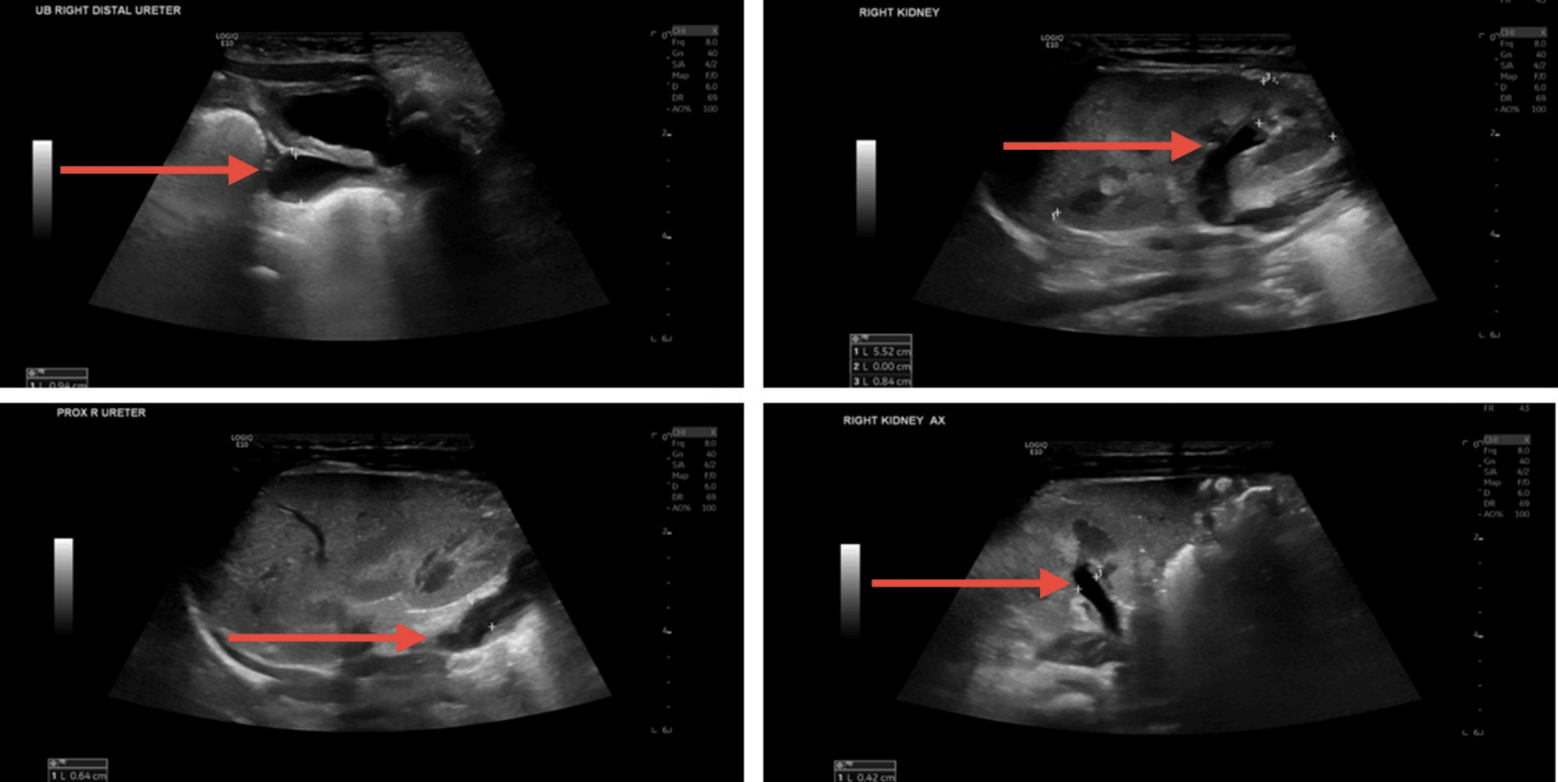
Ultrasound images Images demonstrate right-sided hydronephrosis and hydroureter, suggestive of obstructive uropathy. A dilated renal pelvis and right ureter, extending from the proximal to distal segments, were noted.

Because initial screening had shown a solitary kidney, a follow-up renal ultrasound was performed when the infant was two months old to assess kidney structure and function. The right kidney was normal in size, shape, and echogenicity for age, measuring approximately 5.5 cm in length with preserved corticomedullary differentiation. However, moderate dilation of the right renal collecting system was observed. The renal pelvis was prominent with an anterior-posterior diameter of about 4 mm, and the proximal ureter measured 6 mm in diameter, with the distal ureter up to 9 mm, indicating ureterectasis. These findings corresponded to a postnatal urinary tract dilation (UTD) categorization of UTD P2 (mild hydronephrosis), a condition that had been noted on the initial newborn ultrasound and remained stable (Figure 3).

**Figure 4:**
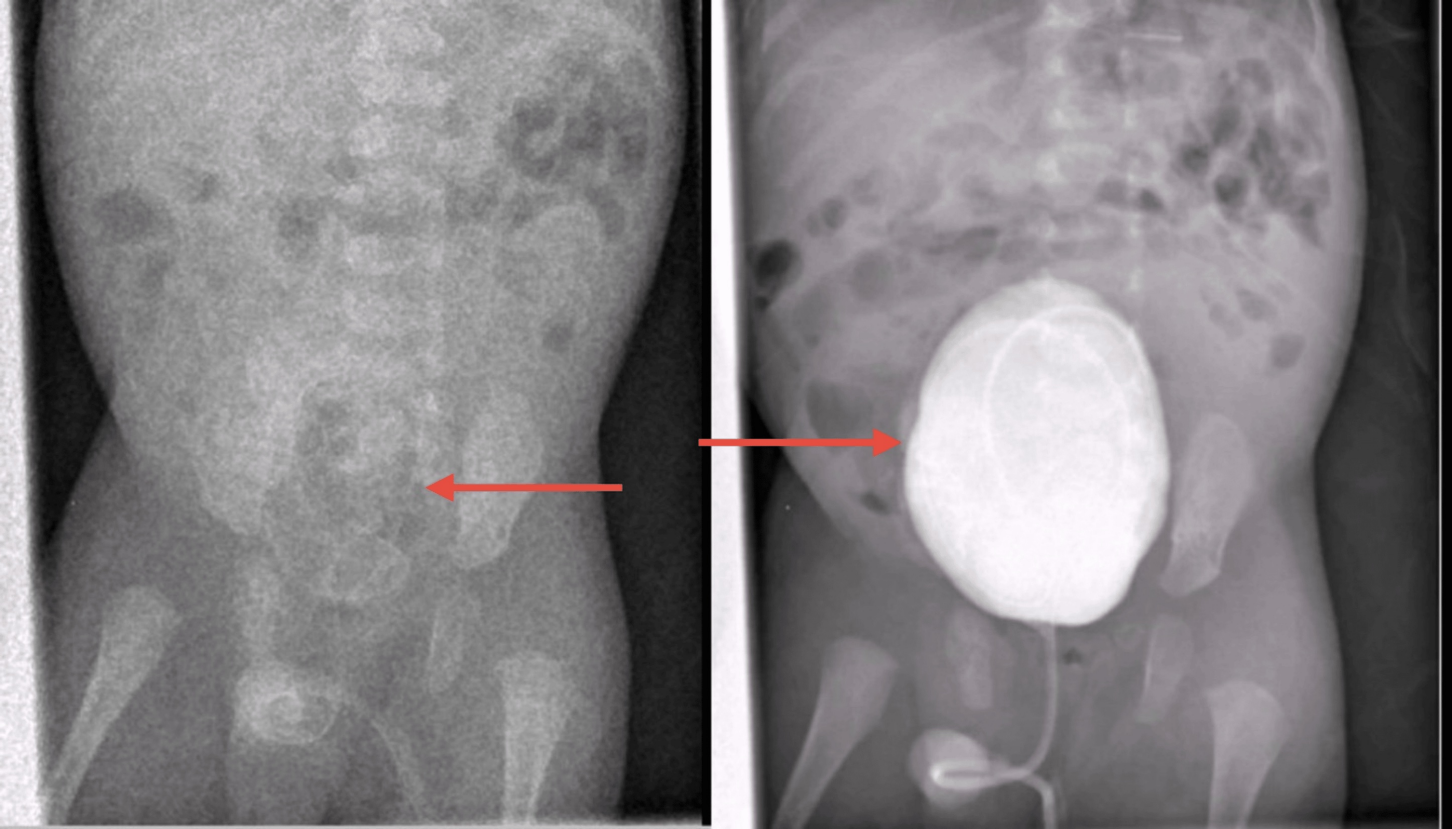
Micturating cystourethrogram (MCUG) images showing a distended, contrast-filled bladder with a Foley catheter in place during the filling phase Grade I vesicoureteral reflux was observed on the right side, limited to the ureter without an involvement of the renal pelvis.

The infant was evaluated by a pediatric urologist, who initiated prophylactic antibiotic therapy to prevent urinary tract infections given the hydronephrosis and potential vesicoureteral reflux. A voiding cystourethrogram (VCUG) was then performed at three months of age to further investigate the urinary tract. The VCUG (Figure 4) revealed a grade I vesicoureteral reflux on the right side.

It also incidentally showed a small bladder diverticulum and a prostatic utricle cyst. There were no radiographic signs of posterior urethral valves or outlet obstruction.

The infant’s care has been coordinated by a multidisciplinary team. To date, the patient has received feeding therapy and uses modified bottles to maintain adequate nutrition. The dermatology team has initiated skin care measures to manage xerosis and prevent secondary infections, while the pediatric urologist continues regular monitoring of the solitary kidney and mild vesicoureteral reflux, with renal growth and function remaining age-appropriate. These ongoing interventions represent essential aspects of current management. In addition, future surgical procedures are planned, including cleft palate repair and reconstructive surgery to improve hand function when the child is older. Pediatric dentistry will continue to monitor dental development given the high likelihood of anomalies in the EEC syndrome. Through this combined approach, both present needs and long-term treatment goals are being addressed.

## Discussion

This case illustrates a classic presentation consistent with EEC syndrome, with additional urinary tract involvement, one of several developmental syndromes caused by mutations in TP63. The TP63 gene is a master regulator of ectodermal and limb development, and its disruption leads to a characteristic constellation of abnormalities [1,2]. As seen in the index case, the combination of ectrodactyly, ectodermal dysplasia features, and cleft lip/palate is the hallmark of EEC syndrome. However, TP63 mutations can produce a wide phenotypic spectrum. Some individuals have more severe skin involvement, such as in AEC (Hay-Wells) or Rapp-Hodgkin syndromes, which feature erosive dermatitis and scalp infections in infancy, whereas others, like the ADULT syndrome, present with limb and ectodermal defects but no orofacial clefting [5,7]. In fact, overlapping features can occur even within the same family. Variability in expression is well documented in TP63 syndromes, as not every patient exhibits all the classic triad features of EEC, and additional anomalies may be present in some cases but absent in others [8] For instance, an individual with a TP63 mutation might have limb malformations and dental anomalies without a cleft palate, whereas a relative with the same mutation could have the full triad. This variability implies that other genetic or environmental modifiers influence the phenotype [10,13].

The specific heterozygous variant identified in this infant, c.740A>G (p.His247Arg), lies in the DNA-binding domain of p63. Missense mutations in this domain are the most common cause of EEC syndrome and typically act in a dominant-negative manner, impairing the ability of p63 to bind DNA and regulate the target genes [1,5]. The His247Arg substitution has been previously reported as a pathogenic change associated with EEC syndrome, including cases with and without orofacial clefts [12]. Our case adds to the phenotypic spectrum linked to this mutation, demonstrating that p.His247Arg can not only cause the prototypical hand/foot and craniofacial anomalies but also congenital kidney and urinary tract defects. Notably, genitourinary malformations are an under-recognized component of EEC syndrome. A review by Hyder et al. (2017) [8] highlighted that a subset of EEC patients have significant genitourinary anomalies, ranging from hydronephrosis and vesicoureteral reflux, as in our patient, to more severe issues like bladder dysfunction or renal dysplasia requiring intensive management. Similarly, a recent prenatal case report from China described an EEC fetus with cleft lip and polycystic kidneys detected on ultrasound [14], and another report documented prenatal detection of nephrogenic cystic changes and hydrops fetalis in an EEC case [15]. In the case of our patient, the solitary kidney and reflux were key findings that required early intervention with prophylactic antibiotics to protect renal function. Such renal and urinary findings reinforce the importance of a thorough systemic evaluation in any infant diagnosed with a TP63 mutation, beyond the immediately obvious limb and facial differences.

The broad range of clinical manifestations in TP63-related disorders has prompted efforts to correlate specific mutations with particular phenotypes. Generally, the EEC syndrome is caused by mutations in the DNA-binding domain (amino acids ~150-350) of p63 that reduce its transactivation capability, whereas AEC (Hay-Wells) syndrome often results from mutations in the SAM (sterile alpha motif) or TI (transactivation-inhibitory) domains of the protein [5,7]. These domain-specific effects can explain why AEC has prominent skin fragility and erosions, while EEC more frequently has limb and craniofacial anomalies. The p.His247Arg mutation lies within the DNA-binding domain and fits the typical EEC mutational spectrum [12]. Interestingly, some TP63 mutations have been observed in multiple syndromes or yield blended phenotypes. For example, the Arg279His mutation in TP63 has been found in classic EEC families and also in patients diagnosed with Rapp-Hodgkin syndrome, which blurs the clinical distinction between those entities [6]. Likewise, an Arg298Gln mutation was identified in the ADULT syndrome but functionally behaves differently from EEC mutations, indicating a gain-of-function effect that spares midfacial development [9]. A report by Alves et al. (2015) [10] described a Brazilian family with a novel TP63 mutation where one member had full EEC syndrome, another had only split-hand/foot malformation, and yet another had features overlapping with the ADULT syndrome, all due to the same genetic change. These observations highlight that while the presence of a TP63 mutation confirms a diagnosis within the p63 syndrome family, the exact clinical course and organ involvement can vary widely. It reinforces the need for individualized assessments of each patient’s manifestations rather than making assumptions based solely on the mutation identified.

Managing EEC syndrome and related TP63 disorders requires a multidisciplinary approach tailored to the patient’s specific set of anomalies [5,11]. With appropriate medical and surgical care, individuals with EEC syndrome can lead healthy lives with near-normal development. Intellectual ability is preserved, and early intervention can mitigate many of the functional impairments caused by the physical anomalies. Our patient’s prognosis is favorable given timely repairs and therapies planned. However, continuous follow-up is necessary, especially through childhood, to manage evolving issues such as dental eruptions or orthopedic challenges as the child grows. From a genetic perspective, this case underscores the importance of family screening once a proband is identified. In our scenario, it immediately allowed for the recognition of the father’s condition and informed the family’s future reproductive choices. Prenatal genetic diagnosis or preimplantation genetic testing can be offered in families with known TP63 mutations, to prepare for or to prevent recurrence [16,17]. Interestingly, emerging research is exploring novel therapies targeting the consequences of TP63 mutations. For example, a unique case of mosaic EEC syndrome enabled researchers to use the patient’s own healthy stem cells to regenerate corneal tissue, improving vision [18]. While such personalized regenerative treatments are not yet generalizable, they represent a hopeful avenue for addressing specific complications of these syndromes. Ongoing studies into p63’s molecular pathways may also yield targeted therapies. For instance, small molecules that could modulate p63 activity or downstream effectors might ameliorate certain features in the future [5].

## Conclusions

This case contributes to the understanding of the EEC syndrome by documenting the clinical course of a child with a heterozygous TP63 (p.His247Arg) mutation inherited from his affected father. The combination of ectrodactyly, ectodermal dysplasia features, cleft lip and palate, and renal anomalies illustrates the broad phenotypic spectrum of TP63-related disorders. Early molecular diagnosis was crucial for confirming the condition and guiding multidisciplinary care. The case also highlights intra-familial phenotypic variability and underscores the importance of genetic counseling for reproductive planning. As no single treatment exists, management should target individual organ involvement. Continued reporting and research into TP63 mutations are essential for enhancing early recognition and optimizing personalized care in these complex developmental syndromes.
